# Genome-scale models as a vehicle for knowledge transfer from microbial to mammalian cell systems

**DOI:** 10.1016/j.csbj.2023.02.011

**Published:** 2023-02-08

**Authors:** Benjamin Strain, James Morrissey, Athanasios Antonakoudis, Cleo Kontoravdi

**Affiliations:** Department of Chemical Engineering, Imperial College London, London SW7 2AZ, United Kingdom

**Keywords:** Mammalian cell metabolism, Resource allocation models, Flux balance analysis, Human pathophysiology

## Abstract

With the plethora of omics data becoming available for mammalian cell and, increasingly, human cell systems, Genome-scale metabolic models (GEMs) have emerged as a useful tool for their organisation and analysis. The systems biology community has developed an array of tools for the solution, interrogation and customisation of GEMs as well as algorithms that enable the design of cells with desired phenotypes based on the multi-omics information contained in these models. However, these tools have largely found application in microbial cells systems, which benefit from smaller model size and ease of experimentation. Herein, we discuss the major outstanding challenges in the use of GEMs as a vehicle for accurately analysing data for mammalian cell systems and transferring methodologies that would enable their use to design strains and processes. We provide insights on the opportunities and limitations of applying GEMs to human cell systems for advancing our understanding of health and disease. We further propose their integration with data-driven tools and their enrichment with cellular functions beyond metabolism, which would, in theory, more accurately describe how resources are allocated intracellularly.

## 1 Introduction

**Fig. 1:**
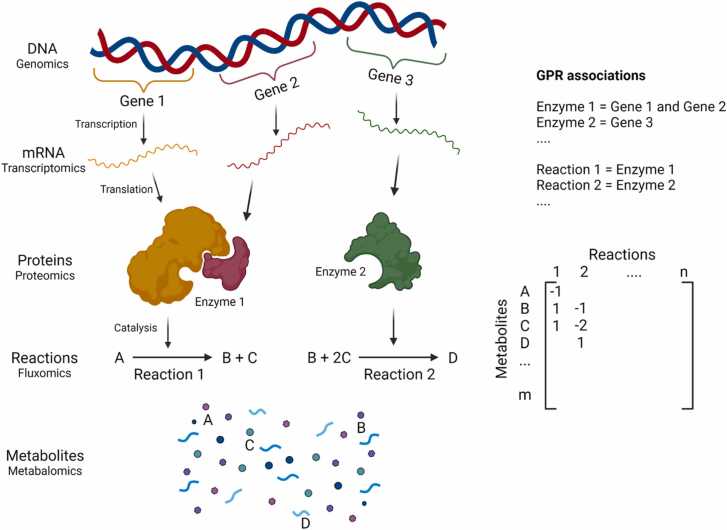
Reconstruction of a generic GEM from the different layers of ‘omics datasets’.

Genome-scale metabolic models (GEMs) are a comprehensive representation of the link between genotype and phenotype, summarising information on genome, proteome and metabolome of a cell [1]. This information is organised in the form of matrices relating genes to metabolic reactions and reactions to metabolites, as well as a set of gene-protein-reaction (GPR) associations [2], [3], as shown in Fig. 1. The construction of the matrices and GPR associations relies on genomic, transcriptomic, proteomic and metabolomic data [4]. Given this information and a set of metabolite uptake/secretion rates that act as constraints, GEMs can calculate the rates of intracellular reactions, thus providing fluxomic information.

GEMs have been constructed for over 6000 organisms [5], [6], including the well-studied *Escherichia coli* [7], *Mus musculus* [8], [9], *Pichia pastoris* [10], *Saccharomyces cerevisiae* [11] and *Homo sapiens* [12], [13], [14], with reconstructions regularly updated to include more complete GPR associations and remove blocked reactions and dead-end metabolites. GEMs can be used to study cell metabolism, optimise bioprocesses, and design strains with enhanced or custom functionality. Historically, GEMs have found greater application in microbial organisms, owing to the smaller model size and relative ease of experimental validation/manipulation compared to mammalian cell systems. The smaller metabolic network size of microbial model systems such as, for example, *E.coli* cells, has also led to the development of a variety of solution methodologies and optimisation algorithms for the design of cells with desired phenotype (summarised in [15]). Although the transfer of the entire repertoire of techniques to mammalian cell systems is often hampered by increased model size and complexity leading to highly underdetermined models, there are already developments in the use of mammalian cell GEMs for strain and process engineering (*e.g.*, [16], [17], [18]), as well as recent algorithm development work applied for understanding the nutritional needs of bioprocessing-relevant organisms but also Atlantic salmon (*Salmo salar*) [19].

In this work, we outline the main remaining challenges in the application of GEMs and related toolkits to mammalian cell systems and zoom in on their application to human cells as a vehicle for understanding health and disease.

## 2 On the use of GEMs for understanding human cell systems

**Fig. 2:**
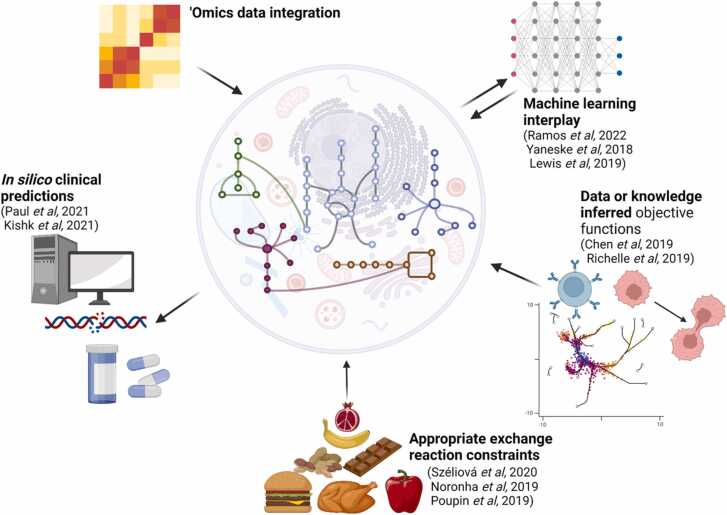
Summary of areas where knowledge can be transferred from mammalian cell GEMs to wider health and disease research.

Advances in clinical sample analysis and *in vitro* disease models are now enabling the generation of similar datasets for human physiology and pathophysiology. It is therefore opportune to examine how learnings and techniques developed for analysing data from biotechnologically relevant organisms using GEMs can be applied in a clinical context to further our understanding of health and disease (Fig. 2). For example, within industry, it is commonplace to generate GEMs specific to a cell line *via* the integration of ‘omics data to prune inactive reactions using techniques such as GIMME [20] (cell line specific model generation is reviewed in depth in [21]). The same holds true in health and disease research, with thousands of patient-derived GEMs having been published for cancer alone [22]. As these models are specific to each disease type, they can be effectively used to explore essential genes in diseased tissue and to identify drug targets. In a recent study, for instance, single-gene knockouts were performed on GEMs of NCI-60 cancer cell line panel to identify and rank genes responsible for the growth of cancerous cells in an effort to identify potential drug targets that would reduce the growth rate of cancer cells but not that of normal cells [23]. This type of analysis is not only limited to chronic diseases; it has also been used in infectious disease studies. In one body of work, flux balance analysis (FBA) was applied to human lung cells infected with severe acute respiratory syndrome coronavirus 2 (SARS-CoV-2) and host-specific essential genes and gene pairs were determined through *in silico* knockouts that were theorised to reduce viral biomass production without affecting the host biomass [24].

These examples highlight the potential of GEMs for analysing large clinical omics datasets in a systematic way that links multiple levels of information. In our opinion, it is possible to envisage the use of GEMs and related methodologies, such as strain design algorithms, in a health setting to generate optimal strategies that reduce disease-associated phenotypes, while improving desirable healthy phenotypes. Herein, we outline the main outstanding challenges towards this end.

### 2.1 Challenges when building mammalian GEMs

Eukaryotes are known to be more biologically complicated that prokaryotes, meaning that applying GEM techniques to eukaryotic organisms is more challenging with respect to obtaining accurate predictions. One of the key sources of difference in complexity between prokaryotes and eukaryotes is the presence of subcellular organelle structures, such as mitochondria, peroxisomes, and nucleus, that do not exist in prokaryotes. Any well annotated eukaryotic GEM must contain these structures and the reactions associated with them for truly accurate predictions [25]. Significantly however, the presence of sub compartments means there is a requirement to gap fill the model using intracellular transport reactions. These transport reactions are often poorly studied and can lead to models with many reversible reactions, which, in turn, may lead to futile cycles, freely exchanging metabolites and protons across compartments, and erroneous energy generation calculations [26]. These cycles have been shown to inflate maximal biomass production rates by 25 % and are known to be present in the majority of published genome scale models [27], with eukaryotic models at greater risk thanks to the increased presence of intracellular exchanges. Ultimately, these results highlight the importance of using an appropriate combination of gap filling algorithms (reviewed in depth here [28], [29]) and manual curation when moving from prokaryotic to more complex, eukaryotic models of metabolism to ensure accurate predictive performance.

While GEMs have been developed for many different species across all domains of life (reviewed in [6]), given the complexity of building eukaryotic GEMs, there has been a lack of regularly updated and publicly curated GEMs for mammalian model organisms such as *Mus musculus* (mouse) and *Rattus norvegicus* (rat) [30]. Instead, recent research developing new modelling techniques using non-human mammalian GEMs has predominantly focused on industrially relevant organisms such as Chinese Hamster Ovary (CHO) cells. To help address this, a framework has recently been published that combines multiple data sources, including the Kyoto Encyclopaedia of Genes and Genomes (KEGG) [31], and generates a coherent collection of GEMs for major model animals using the Human1 GEM as a template [30], [32]. This approach allows for the straightforward development and maintenance of GEMs for multiple species. Since small rodents account for 90% animals used annually in medical research [33], the development of these models using a high-quality model as a backbone opens the possibility to better utilise GEMs in medical research settings, reduce the reliance on model animals and understand differences between model animal and human metabolism.

### 2.2 Determining effective exchange reaction constraints

One of the first challenges that occurs when running GEMs is gathering sufficient extracellular metabolomic data to effectively calculate metabolite uptake rates to constrain the GEM of interest. Within industrial biotechnology, the calculation of these uptake rates is straight-forward, thanks to the relative ease at which extracellular metabolomics can be measured in bioreactors. This means that industrially relevant cultured mammalian cells, such as CHO cell lines, often have detailed constraints for many exchange reactions. This has allowed researchers to understand how the accuracy of this data can affect GEM predictions. For instance, using the CHO cell GEM, researchers have demonstrated that the measurement of low exchange rates of essential amino acids has the biggest impact on the growth rate prediction [34] and that the highly accurate quantification of all uptake and secretion rates was essential for reliable predictions generated by FBA [35].

The generation of such extracellular time-course metabolomics is far more challenging in multicellular organisms. While researchers can culture the cells of interest *in vitro*, this may not be fully representative of how a tissue behaves *in vivo*. This means that generating *in vivo* constraints is of vital importance to accurately understand diseased states, toxicology, and nutrition. A potential method to do this is the use of nutrition databases to calculate the approximate composition of metabolites in a diet that are available for uptake by a cell. One such database is the Virtual Metabolic Human [36], which contains the composition of 11 pre-defined diets that can be downloaded as a flux rate (in mM per person per day). This data can be directly used to constrain the human metabolic model. Significantly, while this resource acts as an excellent baseline for constraining the human GEM to understand differences in diet, given that small changes in the exchange rates of essential amino acids can significantly impact the accuracy of predictions in the CHO GEM [35], it seems unlikely that such a database would provide enough accuracy to consistently give meaningful outputs from a GEM in all use cases.

To overcome this obstacle, techniques that rely on true *in vivo* measurements, such as arterio-venous blood metabolomics (AVBM) profiles, may be considered. In this approach, blood samples are taken from an artery directly before and a vein directly after the tissue type of interest. The difference in metabolite concentrations between these two samples is then presumed to be the amount of metabolite exchanged by the tissue of interest, which can be used to constrain the GEM. This approach has recently been applied to the genome scale modelling of multi-cellular organisms. In one body of work, researchers used AVBM measurements to constrain a GEM to study the global metabolism of liver and intestine of a minipig model of obesity, leading to the identification of upregulated pathways in obese subjects, such as tryptophan metabolism [37]. Nonetheless, while this approach may be appropriate for the genome scale modelling of animal models in the lab, it is highly invasive and unlikely to be acceptable for humans.

### 2.3 Determining appropriate objective functions

The common selection of a biomass maximisation as an objective function for performing FBA of mammalian GEMs is a methodology that largely remains from microbial GEMs, despite that fact it is well known to not be representative of the true ‘objective’ of a mammalian cell, especially outside the exponential growth phase [38]. This lack of suitability of a biomass objective is even more apparent for *in vivo* systems where, unless the tissue of interest is cancerous, cells rarely maximise their proliferation. As a result, researchers trying to model *in vivo* systems must consider the use of alternate objective functions and draw inspiration from mammalian biotechnology solutions. For instance, an unconventional objective function based on the minimisation of non-essential nutrient uptake has been designed for the CHO cell GEM [39]. This method directly estimates essential amino acid uptake fluxes by solving for the “essential minimum” consumption requirements based on cellular growth measurements. This unconventional objective function was shown to distinguish metabolic differences between three distinct CHO cell lines not directly observed using the conventional biomass maximisation. This highlights how the use of more appropriate objective functions may render GEM outputs more information rich, improving their practical application in health and disease research.

The identification of more appropriate objectives may either be achieved applying well established knowledge around the tissues of interest (*e.g.*, a GEM of a B cell may be set to maximise antibody production) or by inferring cell functions through data, such as the analysis carried out by Richelle et al. [40]. In this work, the functions of a cell were inferred from transcriptomics data by considering the gene expression level associated with a metabolic pathway and the number of reactions involved. During this work, a list of tasks was curated resulting in a collection of 210 tasks covering seven major metabolic activities of a cell (energy generation, nucleotide, carbohydrates, amino acid, lipid, vitamin & cofactor and glycan metabolism). These tasks were used to protect selected metabolic features using context-specific model generation algorithms for human, CHO, and mouse cell GEMs. The results highlight that these context-specific models better capture the actual biological variability across cell lines. Similar methodologies can therefore be considered when trying to determine the ‘goal’ of a tissue when selecting an objective function.

In addition to the lack of suitability of maximising biomass, it is important to consider that the biomass formation of mammalian cells is highly variable, depending on factors such as environmental conditions, cell type or culture phase, meaning the biomass equation must be customised for optimal model performance. For example, research in CHO cells has demonstrated cell lines display highly variable total protein content, cell dry mass and lipid composition across cell lines [35]. Moreover, work using the human GEM showed that metabolite composition and associated coefficients of the biomass function had a large impact on the growth rate prediction accuracy of cancer cell lines. In addition, metabolite composition of the biomass equations significantly impacted gene essentiality accuracy [41], meaning a new biomass equation should arguably be determined in each case. To this end, tools originally designed for microbial systems may be used, such as BOFdat [42], to generate custom biomass reactions for mammalian cell systems based on experimental ‘omics data.

### 2.4 On the integration of data-driven modelling with GEMs

In recent years, advances in artificial intelligence and machine learning have revolutionised many areas of biological research [43]. Such approaches have started to be coupled with GEMs to help improve predictions and aid model output analysis. The coupling of GEMs with data- driven methods has been proposed as a method to effectively reduce the solution space by predicting biologically relevant constraints from experimental data (reviewed in depth in [15], [44], [45]). As with the previously discussed methodological areas of genome-scale modelling, this coupling of machine learning with GEMs to improve predictions is at a more advanced stage in biotechnologically relevant mammalian cell systems than it is in human health and disease research. For instance, a recently published method, termed HybridFBA, coupled unsupervised machine learning with a CHO cell GEM. In this approach additional flux constraints were deduced by Principal Component Analysis (PCA) of experimental flux data [46]. Specifically, the authors used each principal component to impose a constraint on the direction of variation of groups of fluxes. This method was shown to significantly improve growth rate predictions compared to standard FBA and was used to design a culture feed *in silico* that led to desired phenotype from target cell lines. This highlights how the coupling of mammalian cell GEMs with machine learning algorithms can improve their performance.

In addition, machine learning methods may be used to better analyse outputs and extract meaning from complex model predictions. For example, flux distribution predictions may be analysed using supervised and unsupervised machine learning methodologies to pick apart key aspects of metabolism that may influence a diseased phenotype of interest. This methodology has already been well applied within health and disease research using GEMs [15], [44], [45]. For example, researchers have used unsupervised learning with GEMs to identify the fluxes that explain most of the data variation in breast cancer patients, reduce dimensionality and create patient groupings [47]. Furthermore, researchers have applied personalised FBA models of patient tumours to predict metabolite production rates. These were input into machine learning classifiers for the identification of metabolite biomarkers associated with radiation resistance. The results demonstrated improved classification accuracy and identification of clinical patient subgroups, marking a significant step toward personalised classifiers for radiation treatment response [48]. These approaches demonstrate the power of using these two techniques synergistically.

## 3 A case for resource allocation models

### 3.1 Benefits of resource allocation

There has been a drive in the systems biology community away from classical stoichiometric network study and towards the study of metabolism through an optimised cellular economy. Resource allocation models (RAMs), as recently reviewed in [49], [50], [51], [52] and with key methods summarised in Table 1, can describe many aspects of metabolism and cellular behaviour [53], [54], where simple stoichiometric balances fall short. So far this drive towards RAMs has been almost exclusively carried out in microbial systems, due to their relative simplicity. Enzyme constrained FBA (ecFBA) models are also considered in this review due to their similarities with RAMs and are included in Table 1.

**Table 1:** Resource allocation models and the current challenges in their application to mammalian systems.

Method	Method Class	Description	Current challenges for application to mammalian systems
FBAwMC [57]	ecFBA	Global constraint on enzyme solvency capacity and kinetics	Achieved already [76]
MOMENT [81]	ecFBA	Inclusion of enzyme concentration in solvency capacity and kinetics	More accurate k_cat_ values, further genome annotation
GECKO [60]	ecFBA	Kinetic and solvency capacity of enzymes with integration of proteomic data	More accurate k_cat_ values, further genome annotation, quantitative proteomic data
RBA [82]	RAM	Inclusion and constraining of translation, replication and transcription machinery	Accurate parameterisation
CAFBA [83]	RAM	Global constraint modelling tradeoff between growth and biosynthetic cost	Accurate parameterisation
ME models [53], [84]	RAM	Addition and coupling of transcription and translation with metabolism	Further genome annotation, quantitative proteomic data, knowledge of expression machinery, computational burden
ETFL [85]	RAM	Integration of expression machinery with thermodynamics	Further genome annotation, quantitative proteomic data, knowledge of expression machinery, computational burden

One key advantage of a RAM approach to metabolic modelling is that the additional constraints greatly reduce the feasible solution space by placing more restrictive bounds on fluxes. This lowers the variability of metabolic fluxes and guides flux towards more biologically feasible solutions. This would be particularly useful for mammalian GEMs, which contain many thousands of reactions [9], [55], [56][refs], and hence have extremely large potential solution spaces.

As well as reducing the solution space of metabolic models, the additional constraints also predict and explain key phenotypes that are not possible with traditional stoichiometric models [57], [58], [59], [60]. Classical models ignore costs related to synthesis and usage of proteins and are limited only by the stoichiometry of metabolites exchanged by the cell with its surroundings, meaning, even if a reaction is unlikely to occur due to the production of an expensive catalysing enzyme, the model is unable to account for this. The communal usage of resources drastically effects the distribution of fluxes through the model. Phenomena such as overflow metabolism do not make sense from a purely stoichiometric point of view and can only be explained in the context of the trade-off between ‘inefficient’ metabolism, protein cost and cell growth [58], [61], [62]. The ability to predict overflow metabolism is an important feature of mammalian cell modelling, such as the Warburg Effect in cancer cells. Being able to better predict peripheral overflow metabolism would be beneficial in the metabolic modelling for clinical research of diseases such as cancer, where non central pathways are known to play a key role [63], [64], [65], [66].

In addition to cancer cell biology, overflow metabolism is important in biopharmaceutical production using mammalian cells *e.g.*, CHO cells. CHO cells typically undergo a lactate-producing phase, in which overflow metabolism is high, followed by a lactate consuming phase as growth rate subsides [67]. The accumulation of lactate is toxic to cell cultures, causing the addition of base to maintain pH set point and subsequently raising osmolality and lowering growth rates [68], [69]. The ability to accurately capture lactate producing and consuming phases through metabolic modelling would aid in process and cell line optimisation.

More recently, other phenomena have been effectively modelling through proteome allocation, for example arginine catabolism in L. lactis [70]. The application of resource allocation to mammalian metabolism would be able elucidate features that have yet to be observed in traditional metabolic modelling.

A further benefit of expanding classical models with resource allocation machinery is the ability to incorporate omics data more effectively. With the increased availability of omics data, GEMs provide an excellent framework for the integration of this data into a combined workflow. As RAMs can consider transcription and translation machinery, transcriptomics and proteomics can be used to constrain metabolism in a more targeted manner, as opposed to current methods which rely on assumptions on the link between reaction rate and gene expression/protein translation [71], [72], [73].

The broadened scope of RAMs allows a more complete understanding of cell behaviour and the relationship between cellular processes. This allows predictions that could not be captured with classical models, such as identifying bottlenecks and gene engineering targets as well as biological parameters *e.g.*, condition-dependent biomass composition [74], [75] and transcription/translation machinery [74].

### 3.2 Challenges in implementation to mammalian systems

While the benefits of RAMs and ecFBA in mammalian systems are numerous, there are obstacles on the path to achieving this goal. One of the main challenges is the scarcity of enzyme data. EcFBA, in particular, rely on the choice of turnover number (k_cat_) values, which are difficult to source for mammalian cells. For example, Yeo et al. were able to find k_cat_ values for 16 % of enzymes in their CHO GEM [76], and several of these were taken from other organisms (*e.g.*, rodent and human) when there was no Chinese hamster data available. Additionally, *in vitro* k_cat_ measurements may differ from those *in vivo*, although the two have been shown to be correlated [77]. These factors render the application of ecFBA to mammalian cell systems difficult and prevent their full utilisation. A potential solution is to use machine learning approaches for k_cat_ prediction [78], which the enzyme amino acid sequences and the structures of their substrates are used to estimate k_cat_ values. Another solution is to infer the apparent k_cat_ value (k_app_) *in vivo*, using measured proteomics and transcriptomics data [77], [79].

A second issue is the aforementioned complexity of mammalian biology compared to simpler systems for which RAMs are more developed. There still exists a knowledge gap for protein sequences and gene-protein-reactions associations in mammalian cells, preventing the construction of effective transcription/translation machinery and integration into metabolism. This could be overcome by considering a reduced system, for example central carbon metabolism, for which biological understanding is more complete. This can then be expanded to consider peripheral pathways when the required data becomes available.

A third issue is the computational burden of fine-grained RAMs. As an example, one of the original *E.coli* RAMs [80], contains around 80,000 reactions from an original GEM of around 2000 reactions. Applying this 40-fold change to Recon 2.2 [55], one of the latest human GEMs, would result in a model of around 300,000 reactions. This makes simulation more computationally expensive, which is particularly problematic for sampling-based approaches. Again, focusing on a reduced system would alleviate this computational burden. Overcoming these challenges is imperative to progress mammalian cell metabolic modelling and to access the benefits that RAMs can offer to the community.

## 4 Concluding remarks

Herein, we summarised the main challenges for applying GEMs and related methodologies to mammalian cell systems, including human cell systems representative of health and disease states. These centred around (a) model size, which makes it cumbersome to apply advanced methodologies and algorithms developed for microbial cell systems in the absence of significant computational power, (b) time course data availability, which may be limited to *in vitro* studies to avoid intrusive sampling, and (c) the choice of appropriate objective functions that are representative of highly specialised human cells. Potential solutions involve (a) the integration of data-driven elements with GEMs, either to derive appropriate constraints that restrict the solution space or to analyse and visualise GEM results, and (b) the development of RAMs for mammalian and, eventually, human cell systems. The flexibility that RAMs offer means that models are widely applicable, beyond exponential cell growth, where traditional metabolic modelling approaches are less effective. The main factors restricting mammalian RAM development include lack of data and, again, computational burden for large models. However, it is possible to make small steps towards the goal of creating full-scale mammalian cell RAMs using microorganism models as inspiration.

## CRediT authorship contribution statement

**BS:** Conceptualization, Investigation, Visualization, Writing – original draft. **JM:** Conceptualization, Investigation, Visualization, Writing – original draft. **AA:** Conceptualization, Investigation, Visualization, Writing – original draft. **CK:** Conceptualization, Investigation, Supervision, Writing – review & editing.

## Conflict of interest

The authors have no conflict of interest to declare.
